# sOCP: a framework predicting smORF coding potential based on TIS and in-frame features and effectively applied in the human genome

**DOI:** 10.1093/bib/bbae147

**Published:** 2024-04-09

**Authors:** Zhao Peng, Jiaqiang Li, Xingpeng Jiang, Cuihong Wan

**Affiliations:** School of Life Sciences, and Hubei Key Laboratory of Genetic Regulation and Integrative Biology, Central China Normal University, Wuhan 430079, Hubei, People’s Republic of China; School of Computer Science, and Hubei Provincial Key Laboratory of Artificial Intelligence and Smart Learning, Central China Normal University, Wuhan 430079, Hubei, People’s Republic of China; School of Computer Science, and Hubei Provincial Key Laboratory of Artificial Intelligence and Smart Learning, Central China Normal University, Wuhan 430079, Hubei, People’s Republic of China; School of Life Sciences, and Hubei Key Laboratory of Genetic Regulation and Integrative Biology, Central China Normal University, Wuhan 430079, Hubei, People’s Republic of China

**Keywords:** small ORF, microprotein, coding potential, genome annotation, machine learning

## Abstract

Small open reading frames (smORFs) have been acknowledged to play various roles on essential biological pathways and affect human beings from diabetes to tumorigenesis. Predicting smORFs *in silico* is quite a prerequisite for processing the omics data. Here, we proposed the smORF-coding-potential-predicting framework, sOCP, which provides functions to construct a model for predicting novel smORFs in some species. The sOCP model constructed in human was based on in-frame features and the nucleotide bias around the start codon, and the small feature subset was proved to be competent enough and avoid overfitting problems for complicated models. It showed more advanced prediction metrics than previous methods and could correlate closely with experimental evidence in a heterogeneous dataset. The model was applied to *Rattus norvegicus* and exhibited satisfactory performance. We then scanned smORFs with ATG and non-ATG start codons from the human genome and generated a database containing about a million novel smORFs with coding potential. Around 72 000 smORFs are located on the lncRNA regions of the genome. The smORF-encoded peptides may be involved in biological pathways rare for canonical proteins, including glucocorticoid catabolic process and the prokaryotic defense system. Our work provides a model and database for human smORF investigation and a convenient tool for further smORF prediction in other species.

## INTRODUCTION

Small open reading frames (smORFs) are unannotated ORFs encoding polypeptides less than 100 amino acids [1], which exist across prokaryotes and eukaryotes, from unicellular bacteria [2], yeast [3], to higher plants [4] and mammals [5]. smORF-encoded peptides (SEPs) are regarded as the major component in a hidden proteome we know very little about, which is far greater than the currently studied proteome [6]. Various essential roles have been identified for SEPs, including hormone signaling activator, cancer inhibitor, chaperone subunits, molecular mimicry and immuno-peptides [7].

Several methods and tools are developed to help researchers identify and validate smORFs with experimental evidence. Translatomic technique ribosome profiling (or Ribo-seq) specifically acquires messenger ribonucleic acid (mRNA) fragments being translated, thus performing well in mining novel smORFs [8]. Tools such as ORF-RATER [9] and RiboTaper [10] are applied to process sequencing data from Ribo-seq to discover novel smORFs. Mass spectrometry (MS) provides more credible traits for identifying SEPs translated by smORFs. Specially designed methods are developed to enrich small proteins [11] and facilitate the detection range for low-abundant SEPs [12]. Additionally, proteogenomic tools are used to generate evidence for novel SEPs from MS data [13].

Besides Ribo-seq and MS experiments, increasing studies on smORFs and SEPs have been fairly relied on studies *in silico*, including bioinformatic databases and tools. Numerous smORFs with experimental evidence are collected to provide integrated information in databases such as SmProt [14], sORFs.org [15] and OpenProt [16]. There are mainly two bioinformatic ways to identify translatable smORFs: alignment-based and intrinsic feature–based. The early methods are mainly based on sequence alignment, such as BLASTX search in the software CPC [17], annotating family members in SPADA [18], phylogenic conservation scoring in PhyloCSF [19]. However, alignment-based methods are limited to conservation and perform weakly in exploring unannotated genes, which is predominant in smORF prediction [20].

Recently, methods evaluating coding potential based on sequence intrinsic features have been developed to predict smORFs from long non-coding RNAs (lncRNAs) and other transcripts. For instance, the sORF finder evaluates coding potential according to the nucleotide composition bias as well as synonymous and non-synonymous rate [21]. The CPAT uses ORF length, ORF coverage, Fickett score and Hexamer usage bias to distinguish coding RNAs from non-coding RNAs (ncRNAs) [22], and MiPepid applies the 4-mer feature to train a logistic regression (LR) model [23]. CPPred distinguishes coding RNAs and ncRNAs based on support vector machine (SVM) classifier and sequence features of RNA [24], while DeepCPP uses a conventional neural network (CNN) model [25]. The csORF-finder evaluated the coding potential of sORF in multiple species with in-frame sequence features [26]. Besides methods aimed at human and other mammals, the smORF prediction also goes ahead in plants, such as sORFPred [27], and in prokaryotes, such as PsORFs, sORFPredictor and ProsmORF-pred [28–30].

However, except for the early sORF finder, most methods are designed for predicted smORFs with a pre-trained model, thus incapable of screening candidate smORFs from genomes of other species, which is a demand for genome annotation and data mining in current studies. Here, we constructed the sOCP framework for exploring novel smORFs, from making the smORF coding potential prediction model to building a smORF database from the genome. Our model in human surpassed previous methods in prediction for the testing dataset and an irrelevant dataset, OpenProt Altprots. Finally, we used the model to predict novel smORFs from the human genome with different start codons and generated the smORF database with high confidence. Annotation of the SEPs suggested their unique characteristics in biological functions. Our work provides a model to predict smORFs in human, mines a large number of novel smORFs and proposes a useful tool to predict smORFs in other species.

## MATERIAL AND METHODS

### Data collection and preprocessing

The positive and negative datasets were generated to construct and evaluate the predicting model. For positive datasets, 114 132 human mRNAs from National Center for Biotechnology Information (NCBI) RefSeq and OpenProt Refprots were integrated together. 66 914 mRNA ORFs from NCBI RefSeq as described in CPPred [24]. We also retrieved 110 710 mRNA ORFs from OpenProt RefSeq. The mRNA ORFs from both resources were integrated after the redundancy removal using the software CD-HIT [31] with the cutoff parameters ‘-c 0.9 -s 0.9’. The nucleotides at the upstream three sites (−3, −2 and −1) were added to the head of each coding sequence (CDS) for feature extraction. Afterward, 8394 ORFs with no more than 101 codons were selected from the total mRNA dataset as the smORF mRNA dataset, and these smORFs were used as the positive dataset in the following procedure.

For the negative dataset, we retrieved 67 885 human ncRNAs from the Ensemble database (release 109) and extracted the maximum ORFs within the ncRNAs [32]. 57 260 ORFs with no more than 101 codons were chosen as the ncRNA dataset. To balance the ratio of the positive and negative datasets, we randomly selected 8394 samples from the ncRNA dataset as the negative dataset.

The positive and negative datasets were split separately for training and testing at a rate of 2:1. The redundant sequences between the training and the testing dataset are removed using CD-HIT with the cutoff parameters ‘-c 0.9 -s 0.9’. The positive and negative training dataset was then split for the following 10-fold cross-validation (CV).

The predicted human smORFs and their evidence information for the sOCP model evaluation were retrieved from OpenProt Altprots, with a size of 453 531 and a length of less than 101 codons. The human genome sequence and annotation files were retrieved from the NCBI genome database (version GRCh38.p14, committed by Genome Reference Consortium). The canonical protein sequences in the human proteome were retrieved from the UniProt database, with the accession ID UP000005640_9606 [33].

### Sequence encoding

To encode an ORF sequence with the upstream three nucleotides into a numerical vector used for coding potential prediction, we extracted sequence features of four classes: the basic features (ORF length, 1-mer and 2-mer), the translation initiation site (TIS)–related features (nucleotide bias), the in-frame features (hexamer score, codon bias, Fickett score) and the continuous and discontinuous *k*-mers (3-mer, 4-mer, 1-gap, 2-gap, 3-gap, 1-bigap, 2-bigap, 3-bigap). Most of the feature extraction methods referred to the description in DeepCPP [25], except for the following two features.

Nucleotide bias is calculated as a 6-dim vector instead of a summation:


$$ {\left[ Nucleotide\ bias\right]}_i=\mathit{\log}\frac{p_{pos}\left({x}_i\right)}{p_{neg}\left({x}_i\right)},x\in \left\{A,C,G,T\right\},i\in \left\{-3,-2,-1,4,5,6\right\} $$

where *i* denotes the six sites around the start codon and *p*(*x_i_*) denotes the probability of *x* at site *i* according to the positive or negative dataset.

Codon bias is calculated as follows:


$$ Codon\ bias=\frac{1}{n}\sum_{i=1}^n\mathit{\log}\frac{p_{pos}\left({C}_i\right)}{p_{neg}\left({C}_i\right)} $$

where *C_i_* denotes the *i*-th codon in the ORF sequence, and *p*(*C_i_*) denotes the codon usage bias of *C_i_* according to the positive or negative dataset, which is the ratio of the codon count to its encoding amino acid count.

### Model selection

The LR, SVM, random forest (RF) and gradient tree boosting (GTB) models were constructed using the Python package scikit-learn [34]. The CNN model was built using the Python package keras (https://keras.io/). The model performance was evaluated through a 10-fold CV.

### Feature ablation

The total extracted features from the ORF sequence were merged into a complete 1169-dim vector and divided into nine components: 3-mer, 4-mer, 1-gap, 2-gap, 3-gap, 1-bigap, 2-bigap, 3-bigap and the others (including the basic, TIS and in-frame features). Nine feature subsets were generated, where each subset one of the nine components was missing. Then, the feature subsets were used in a 10-fold CV for evaluation, and the best-performing one would be retained for the next step. After that, eight feature subsets were generated and evaluated as above. The ablation experiment would go on step by step to determine how the model’s performance altered as the most inefficient feature components were discarded one by one.

### mRMR-IFS

The feature selection method mRMR-IFS consists of two steps: mRMR (maximum relevance minimum redundancy) ranking and IFS (incremental feature selection). The feature ranking was implemented using the Python package pymrmr with the scheme MIQ (mutual information quotient) [35]. For the IFS procedure, *n* feature subsets were generated for the original *n* features, and in the *i*-th subset, only the top-ranking *i* features would be retained. Then all the feature subsets were evaluated in a 10-fold CV, to show the alteration of the model performance as the corresponding features increased from the top ranking 1 to *n*.

### Performance evaluation

For comparing performance evaluation among different methods, ORF sequences with the upstream three sites were provided to CPPred, DeepCPP and sORFPredicor, and ORF sequences without the upstream nucleotides to csORF-finder.

The model performance was evaluated using the following metrics: SN (sensitivity/recall), SP (specificity), PRE (precision), ACC (accuracy), F1S (f1-score), HM (harmonic mean of SN and SP) and MCC (Matthew’s correlation coefficient), calculated as follows (TP: true positive, TN: true negative, FP: false positive, FN: false negative):


$$ SN=\frac{TP}{TP+ FN} $$

$$ SP=\frac{TN}{TN+ FP} $$

$$ PRE=\frac{TP}{TP+ FP} $$

$$ ACC=\frac{TP+ TN}{TP+ TN+ FP+ FN} $$

$$ F1S=\frac{2\ast PRE\ast SN}{PRE+ SN} $$

$$ HM=\frac{2\ast SP\ast SN}{SP+ SN} $$

$$ MCC=\frac{TP\ast TN- FP\ast FN}{\sqrt{\left( TP+ FP\right)\ast \left( TP+ FN\right)\ast \left( TN+ FP\right)\ast \left( TN+ FN\right)}} $$

The cutoff of the classifier was increased stepwise from 0 to 1 to generate the ROC (receiver operating characteristic) curve, with corresponding false positive rate (FPR), equal to 1-SP and true positive rate (TPR), equal to SN) as *x* and *y* coordinates. The area under the curve (AUC) was applied as an evaluation metric. The precision-recall (PR) curve was plotted in a similar procedure, using precision (PRE) and recall (SN) as *x* and *y* coordinates instead.

### smORF prediction in the human genome and annotation

The human genome assembly is scanned for the sense and anti-sense strands to search for smORFs with no more than 101 codons. Non-ATG start codons will also be considered (CTG, GTG, ACG, TTG, ATT, ATC, ATA, AAG, AGG). For each smORF (with the upstream three sites), the sequence features are extracted for the sOCP model to predict a score. According to the start codon, a different cutoff is applied to filter out smORFs. Then, the smORFs located within canonical CDSs are removed. In the end, the smORF sequence FASTA file, the annotation BED file and the information file (recording the upstream three sites, the overlapping gene) are generated to form a novel smORF database in human.

For annotation, smORFs were first encoded into protein sequences using SeqKit [36], and all non-ATG start codons were changed to methionine manually. Then, these SEPs were annotated using the online resource eggNOG-mapper [37]. The canonical proteins in the human proteome were annotated as well for comparison. The protein numbers in each Clusters of Orthologous Groups (COG) category, Gene Ontology (GO) term and Kyoto Encyclopedia of Genes and Genomes (KEGG) pathway were then collected from the annotation file. The standardized ratio is calculated as follows:


$$ Standardized\ ratio=\frac{S_i}{P_i}\ast \frac{P_{anno}}{S_{anno}} $$

where *S_i_* and *P_i_* denote the number of SEPs and canonical proteins in COG category *i* and *S_anno_* and *P_anno_* denote the number of total annotated SEPs and canonical proteins.

## RESULTS

### The framework of sOCP model construction

**Figure 1:**
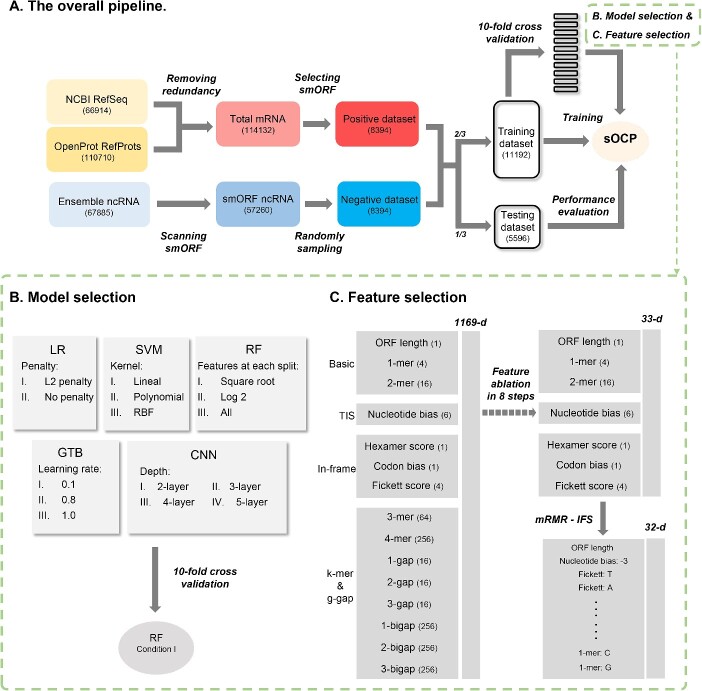
Construction of the sOCP model. The overall pipeline is shown in (**A**), and details are described in (**B**) model selection and (**C**) feature selection.

To construct an sOCP model in human, we first collected and processed mRNAs from NCBI RefSeq and OpenProt RefProts and ncRNAs from Ensemble (Figure 1A). We selected 8394 smORFs as positive samples from mRNAs and the same number of smORFs from ncRNAs as negative samples. The samples were then divided into training and testing datasets. The training dataset was applied in a 10-fold CV to process the model and feature selection. The RF method was verified to surpass other algorithms in model selection (Figure 1B). After feature ablation and mRMR-IFS, we determined a 32-d feature vector to construct the model (Figure 1C). The completed model was evaluated to perform well by the testing dataset and further by the predicted smORFs in OpenProt AltProts.

### TIS and in-frame feature exploration

**Figure 2:**
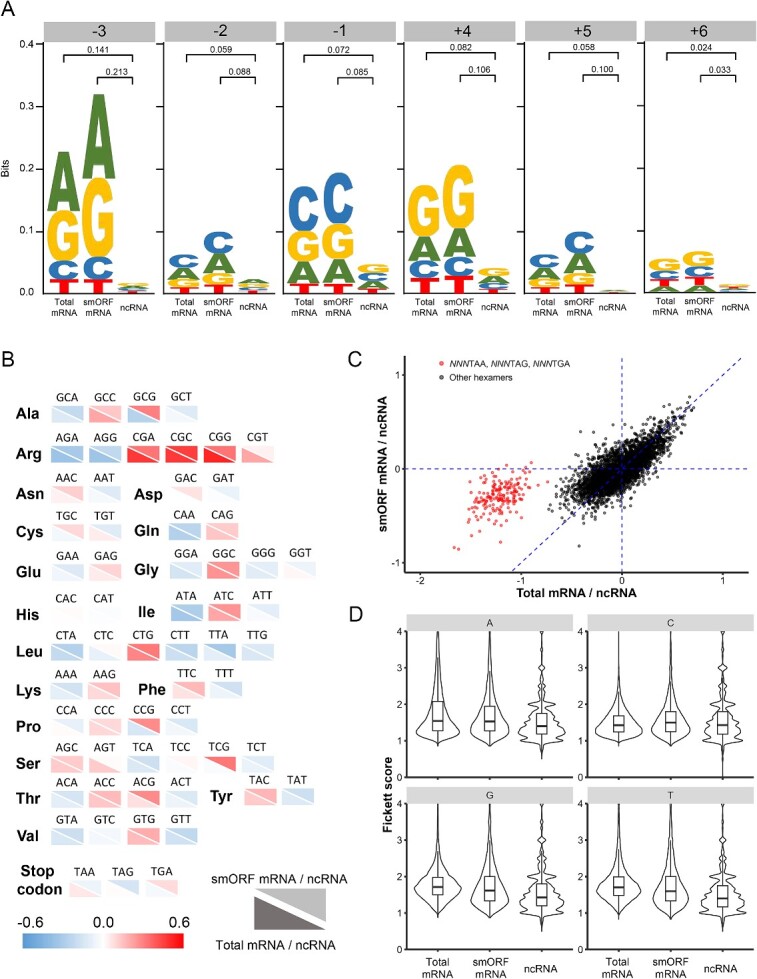
TIS and in-frame features among total mRNA, smORF mRNA and ncRNA. (**A**) The sequence logo representation at site −3, −2, −1, 4, 5 and 6. The number above between the two datasets indicates the KL divergence of the distribution for the datasets. (**B**) The codon bias representation. (**C**) The comparison of the ratio of hexamer scores between total mRNA/ncRNA and smORF mRNA/ncRNA. (**D**) The distribution of Fickett scores for the four nucleotides.

ORF length and *k*-mers are the commonly used characteristics of nucleotide sequences. Besides that, we expected to explore TIS and in-frame features by comparing mRNA and ncRNA, as well as the smORF subset and the total mRNA dataset. We first inspected the nucleotide bias around TIS, including sites −3, −2, −1, 4, 5 and 6. The sequence logo shows a low height for ncRNA at all sites, indicating an even distribution of nucleotides (Figure 2A). The high symbols were observed for mRNA, suggesting a biased distribution, especially for sites −3, −1 and 4. However, the symbol for the smORF mRNA dataset was relatively higher than the total dataset, indicating that features extracted from the smORF subset were distinguishable. Consistently, higher Kullback–Leibler (KL) divergences were observed between the smORF subset and the ncRNA dataset. Moreover, as the nucleotide biases varied widely among the sites, we decided to consider the feature for each site separately.

The in-frame feature codon bias stands for the usage frequency of a codon for its coding amino acid. After visualizing the differences in codon bias between mRNA and ncRNA, we found that except for a few codons, such as CAC and CAT coding histidine (His), there was an apparent distinction between mRNA and ncRNA (Figure 2B). For example, high ratios were observed in CGA, CGC, CGG and CGT among the codons for arginine (Arg), indicating that mRNA tended to use these codons. Although in most of the codons, the ratios were similar for the smORF subset and total mRNAs, it was seen that the smORF mRNAs dataset had its specific codon bias compared with the total mRNAs, such as GCG coding alanine (Ala), TGC and TGT coding cysteine (Cys), CCG coding proline (Pro) and stop codons.

Hexamer score, another in-frame feature, reflects the overall usage of in-frame 6-mers. It is calculated by summing the logarithms of the mRNA/ncRNA likelihood ratio for the hexamers in the smORF. We found that some hexamers had different frequencies between mRNA and ncRNA (Figure 2C). Another invoke result was that the hexamers with a stop codon stayed clearly apart and more distinct in the *x* direction. It was concluded that the usage of the hexamers at the end of mRNA differed from that of ncRNA, and the difference was slighter in smORFs.

We also examined the Fickett score, which represents the position preference of a nucleotide in in-frame 3-mers. For all the four nucleotides, the Fickett scores of mRNAs were higher than those of ncRNAs, showing that the frequency of nucleotides among the three positions was more characteristic in mRNA (Figure 2D). Moreover, the smORF subset tended to be similar to the total dataset in the distribution of Fickett scores.

### Model and feature selection for sOCP

**Figure 3:**
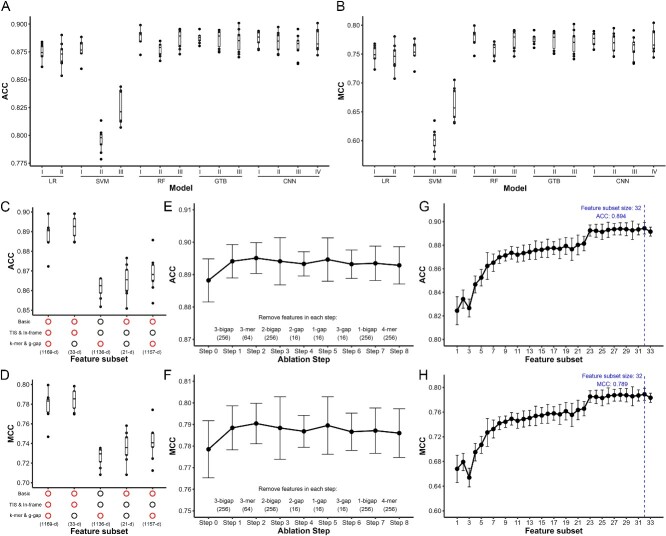
Model selection and feature selection. The comparison of (**A**) ACC and (**B**) MCC among different methods and hyperparameters. The comparison of (**C**) ACC and (**D**) MCC among different feature subsets. The alteration of (**E**) ACC and (**F**) MCC in the feature ablation steps. The alteration of (**G**) ACC and (**H**) MCC in the mRMR-IFS steps. The vertical dash line indicates the best-performing feature subset.

After TIS and in-frame features were testified to be distinguishable, we combined those features with commonly used *k*-mers and g-gaps (discontinuous *k*-mers) (Figure 1B) to run model selection. We compared the popular methods, including LR, SVM, RF, GTB and CNN, with two to four adjusted main parameters for each method (Figure 1C). The RF method with the parameter max_features setting as ‘sqrt’ performed best among all methods, both in the evaluation metrics ACC (0.888) and MCC (0.779) (Figure 3A and B). Hence, the sOCP model in human was based on the RF method in the following procedure.

A complicated feature vector as long as 1169 would probably cause overfitting since the positive and negative datasets contained just thousands of smORFs. More than that, such a long vector would be unacceptably time-consuming to predict millions of smORF candidates in the genome. Therefore, we attempted to shorten the feature vectors by picking essential features from the total features. We first focused on *k*-mers and g-gaps, which took over a large proportion of vector length. We divided features into three subsets: basic features, TIS and in-frame features, *k*-mers and g-gaps. By evaluating the model using different combinations of the subsets, we found that *k*-mers and g-gaps were poorly efficient as a whole, while TIS and in-frame features were important for the model performance as expected (Figure 3C and D). Thus, an ablation experiment was carried out step by step to see if some individual features among *k*-mers and g-gaps would contribute to the model indispensably. However, only tiny fluctuations of the evaluation metrics were observed in the ablation steps (Figure 3E and F). As a result, all eight features, namely, 3-mer, 4-mer, 1-gap, 2-gap, 3-gap, 1-gap, 2-bigap and 3-bigap, could be removed without efficiency loss to simplify our model greatly.

For the remaining 33-dim feature vectors, a further feature selection was designed using the mRMR-IFS method. All the feature components were ranked by the mRMR algorithm first by maximizing relevance and minimizing the redundancy of the features (Table 1). Then, starting from the feature subset containing only the ranking first feature, the feature subset size increased as the top-ranking features were added one by one. All 33 subsets during the incremental step were evaluated, showing that the feature subset with 32 features reached the peak in both ACC (0.894) and MCC (0.789) (Figure 3G and H). Above all, we decided to use the 32-dim feature vectors to serve our model.

**Table 1:** mRMR feature ranking

Ranking	Feature	Ranking	Feature	Ranking	Feature
1	ORF length	12	2-mer: AT	23	Hexamer score
2	Nucleotide bias: −3	13	2-mer: GC	24	Codon bias
3	Fickett: T	14	2-mer: GG	25	Nucleotide bias: −2
4	Fickett: A	15	2-mer: GT	26	Nucleotide bias: −1
5	Fickett: C	16	2-mer: TC	27	2-mer: AC
6	Fickett: G	17	2-mer: TG	28	2-mer: AA
7	2-mer: CG	18	2-mer: CA	29	Nucleotide bias: 6
8	2-mer: GA	19	1-mer: T	30	1-mer: A
9	2-mer: CC	20	2-mer: AG	31	1-mer: C
10	2-mer: TA	21	Nucleotide bias: 4	32	1-mer: G
11	2-mer: CT	22	Nucleotide bias: 5	33	2-mer: TT

### Evaluation of the sOCP model

**Figure 4:**
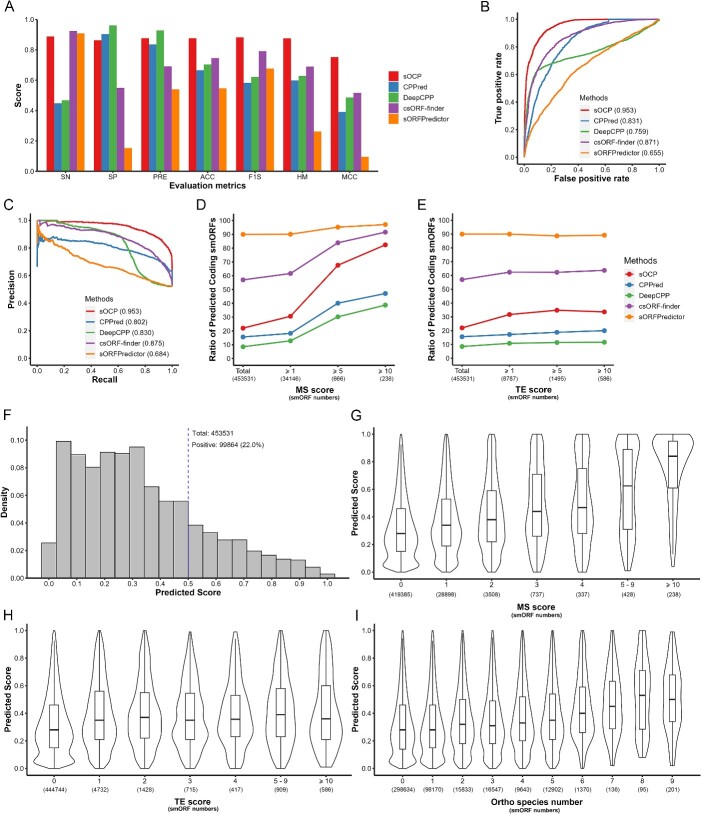
Evaluation of the sOCP model. The comparison of sOCP with CPPred, DeepCPP, csORF-finder and sORFPredictor on (**A**) different metrics, (**B**) ROC curve and (**C**) PR curve for the testing dataset. The comparison of sOCP with CPPred, DeepCPP, csORF-finder and sORFPredictor on correlation to MS (**D**) and TE (**E**) score for OpenProt dataset. (**F**) The density plot of sOCP predicted scores for the OpenProt dataset. The vertical dash line indicates the default cutoff for prediction. The distribution of sOCP predicted scores according to MS score (**G**), TE score (**H**) and Ortho species numbers (**I**).

According to the above results, the sOCP model in human was constructed based on the RF method and the determined feature subset. To evaluate the performance of the sOCP model, we first compared it with four previous models (CPPred, DeepCPP, csORF-finder and sORFPredictor), by predicting the coding potential of smORFs in the testing dataset (Figure 4A). It was shown that for SP and PRE, sOCP was comparable with csORF-finder and sORFPredictor and performed much better than CPPred and DeepCPP. While for SN, it was the reverse. It was referred that sOCP surpassed others by meeting the standard of both specificity and sensitivity in prediction. Consistently, for the overall metrics integrating both SP/PRE and SN, such as ACC, FIS, HM and MCC, the sOCP model scored around 0.8, significantly higher than others. The ROC and PR curve, which considers the model performance with ranged cutoffs, were also shown. The sOCP was above other methods and possessed a larger area under the curve (AUC), as 0.964 for ROC and 0.961 for PR (Figure 4B and C).

Since the testing dataset was to some extent homogeneous with the training data for the sOCP model, we further processed the performance evaluation on the smORF dataset from OpenProt Altprots, which was predicted *in silico* and correlated with translatomic and proteomic evidence (TE and MS score). The ratios of coding potential smORFs were compared among different subsets, from the total predicted smORFs to highly confident smORFs with an MS score of no less than 10. The sOCP model significantly clarified different datasets (Figure 4D). While about 20% of the total smORFs were predicted to be positive, the proportion increased to 67.6% for the smORFs with an MS score of no less than 5 and 82.3% for the smORFs with an MS score of no less than 10. The other methods were testified as well. CPPred and DeepCPP predicted a similar ratio of coding potential smORFs with sOCP for the total dataset but only found around 40% for the smORFs with MS score no less than 10. On the contrary, csORF-finder and sORFPredictor predicted more than 90% of smORFs with MS score no less than 10 but classified most of the total predicted smORF as coding potential. The TE score was used the same as the MS score and received a less apparent result (Figure 4E). As predicted by sOCP, the ratio increased from 20% for the total dataset to 30% for the smORFs with TE scores more than 0. However, the other methods hardly distinguished the smORFs with TE score, except for the csORF finder with a ratio increase of about 5%.

Since the coding potential smORFs predicted by sOCP were proved to be relevant to the evidence, we checked the relationship between the exact sOCP scores and the evidence. About 22% of smORFs were predicted to be positive, while the density of positive smORFs decreased as the score increased (Figure 4F). As the MS score increased, the smORF dataset showed an uprising of the distribution of predicted scores (Figure 4G), indicating a significant correlation between the sOCP score and MS evidence. A similar correlation was also observed for Ortho species number (the number of species in which a homologous smORF is predicted in the OpenProt database) (Figure 4I). However, for the TE score, which is less solid evidence compared with the MS score, the increase was only between zero and non-zero (Figure 4H).

As several methods have been developed previously, the sOCP pipeline meets the demand for current research on smORF and shows novel characteristics in this application. First, the existing methods are limited to several model organisms or integrate data from different species. Tools are needed to help researchers use species-specific data to construct a machine-learning model that can predict the coding potential of smORFs in a species of interest. Therefore, sOCP provides a series of functions for model construction, including dataset processing, feature extraction, parameter optimization and performance evaluation. Besides human, the sOCP pipeline has been also applied in rat (*Rattus norvegicus*) as an example. Based on the datasets from NCBI and Ensemble, we applied the basic, TIS and in-frame features and RF method in training using the sOCP pipeline and got a model with ACC 91.2% and MCC 82.4%. The rat smORF model is provided within the sOCP and can be improved further using feature selection and model selection in the sOCP pipeline. Second, methods such as CPPred and DeepCPP mainly focus on RNA sequences. However, smORF candidates from genomes or *de novo* proteomics are in the form of ORFs, which are partial RNA sequences and might possess different features. The sOCP model is developed to seize the intrinsic features of ORFs and constructed based on a feature vector with fewer dimensions compared with hundreds of dimensions in complicated models provided by DeepCPP or sORFPredictor. This simple model has been proven to be in line with the proteomic evidence of smORF candidates in OpenProt. Third, the sOCP pipeline can be used to build an smORF database for species of interest. To discover smORFs in proteomics studies, a six-frame translation of the genome is too large for efficient database search. Using sOCP, researchers can build a database with coding potential smORFs from the genome. The non-canonical start codons are frequently found in smORFs and have been considered in the updated tool of CPPred, CPPred-sORF [38], which used the non-canonical start codons to distinguish small coding RNAs and lncRNAs. In the sOCP pipeline, CTG or other frequently non-ATG start codons are also applied to predict smORFs in the genome.

### Prediction of novel human smORFs

**Figure 5:**
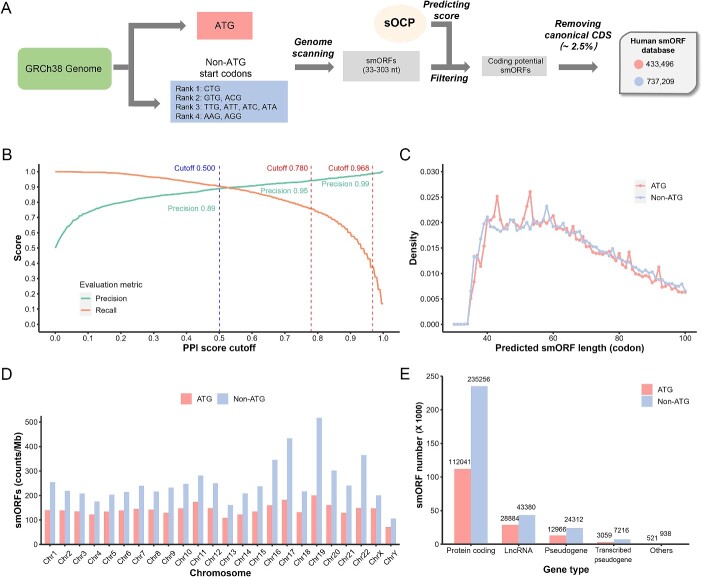
Predicting coding potential smORFs from the human genome. (**A**) The pipeline of prediction. (**B**) The selected score cutoffs for prediction according to evaluation metrics. (**C**) The distribution of smORFs according to their length. (**D**) The distribution of smORFs according to their chromosome locations. (**E**) The distribution of smORFs according to their overlapping gene types. ATG and non-ATG codons are indicated with different colors.

We finally used the sOCP model to predict novel smORFs from the human genome. A pipeline was designed consisting of scanning smORFs with an optional start codon and limited length, predicting the coding potential of the smORFs and removing the smORFs belonging to canonical CDSs (Figure 5A). We retrieved the chromosome sequences and annotations from the GRCH38.p14 assembly. First, the chromosome sequences were scanned by the criteria below: a start codon at the beginning, either ATG or non-ATG; a standard stop codon; and a length between 11 and 101 codons. The scanned smORFs were then predicted by sOCP and filtered according to the corresponding cutoff discussed below.

To build a database of highly credible smORFs, we applied a stringent cutoff in the prediction to increase the precision at the risk of recall (Figure 5B). Non-ATG start codons have been reported to be widely distributed in smORF, and these codons were considered and ranked based on their efficiency and frequency in previous research [39, 40]. For ATG and the most efficient and frequent (Rank 1) non-ATG start codon CTG, the cutoff was set as 0.780 to increase precision from 0.89 (at cutoff 0.5) to 0.95 (Table 2). For Rank 2 and 3, the cutoff was set as 0.968 and 1.000. Rank 4 codons AAG and AGG were all filtered out. The prediction step reduced the number of predicted smORFs from about 38 million to 1,201,397. Afterward, about 2.5% of the smORFs located within canonical CDSs were removed, resulting in 1 170 705 left. The final smORF dataset contained 433 496 smORFs started with ATG and 737 209 with non-ATG start codons.

**Table 2:** Selecting cutoffs in smORF predicting for different start codons

Start codon	Cutoff	Precision	smORF number
ATG	0.780	0.95	433 496
Non-ATG			737 209
CTG	0.780	0.95	695 054
GTG	0.968	0.99	25 806
ACG	0.968	0.99	5499
TTG	1.000	1.00	2407
ATT	1.000	1.00	2994
ATC	1.000	1.00	3310
ATA	1.000	1.00	2139
AAG	–	–	–
AGG	–	–	–
Total			1,170,705

We then inspected the characteristics of the smORFs predicted from the human genome. First, the length distribution showed that most of the smORFs ranged from 40 to 60 codons, and the density decreased as the length increased to 101 (Figure 5C). Second, the distributions on different chromosomes were also shown (Figure 5D). As the smORFs started with ATG had slight variation, the smORFs with non-ATG start codons had a significantly high density in several chromosomes. It suggested an undiscovered trait in the codon composition at some coding areas of these chromosomes. Finally, we explored if the predicted smORFs overlapped with annotated gene locations (Figure 5E). About 40% (468 633) of smORFs overlapped with locations annotated as genes. The majority (74%) of overlapping genes were protein-coding, followed by lncRNA (15%), pseudogenes (8%) and transcribed pseudogenes (2%).

### Annotation of predicted smORF-encoding proteins

**Figure 6:**
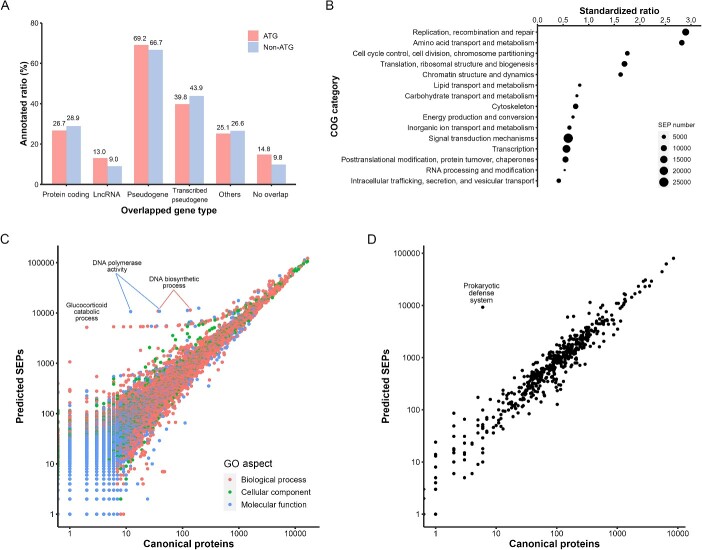
Annotation of predicted SEPs and comparison with the canonical proteins. (**A**) The annotated ratio of predicted SEPs according to their overlapping gene types. ATG and non-ATG codons are indicated with different colors. (**B**) Standardized ratio of predicted SEP number to canonical protein number for COG categories. The point size indicates the SEP number. (**C**) Comparison of predicted SEP number with canonical protein number for GO terms. The colors indicate three different aspects of the GO terms. (**D**) Comparison of predicted SEP number with canonical protein number for KEGG pathways.

To investigate the characteristics of the predicted smORFs, we further translated them into SEPs. As Met-tRNA_i_^Met^ is generally used in initiation for non-ATG translation [39], all non-ATG start codons were encoded into Met as well. 1 170 705 SEP sequences were annotated based on homology, generating 218 709 annotation results (Figure 6A). We observed that SEPs overlapping with pseudogenes obtained the highest annotated ratio, showing that more than 60% were homologous with canonical proteins. Only a quarter of SEPs overlapping with protein coding regions were annotated, probably because after removing CDS-covering smORFs, these SEPs mostly overlapped with untranslated regions (UTRs) or introns within the global protein-coding regions. It was as expected that SEPs overlapping with lncRNAs or not overlapping had a lowest annotated ratio.

We applied the annotation of canonical proteins in the human proteome as a control to figure out the unique features of predicted SEPs. Using the number of canonical proteins in each COG category for standardization, we found that SEPs tended to located in categories ‘Replication, recombination and repair’ and ‘Amino acid transport and metabolism’, suggesting their enrichment in the metabolism of DNA and amino acid (Figure 6B). Besides, there were more SEPs in categories related to cell cycle and translation and less SEPs in categories related to signal transduction and RNA metabolism. For more details on function, we compared SEPs and canonical proteins in GO terms and KEGG pathways (Figure 6C and D). A 10:1 ratio was seen for the majority of terms and pathways, equal as the ratio of 218 079 annotated SEPs to 20 112 canonical proteins in total. However, several outliers indicated specific functions enriched in SEPs while rare in canonical proteins. For example, there were only two canonical proteins in the glucocorticoid catabolic process (GO:0006713). However, as many as 5188 SEPs participated in this biological pathway and possessed 11-beta-hydroxysteroid dehydrogenase activity (GO:0003845), which modifies actions of glucocorticoids and thus maintains the body homeostasis [41]. Moreover, 10 784 SEPs were related to DNA polymerase activity (GO:0034061), with 39 canonical proteins in the same GO term. Similarly, 11 417 SEPs were related to DNA biosynthetic process (GO:0071897), with 137 canonical proteins in the same GO term. The DNA polymerase activity and DNA biosynthetic process were both involved in DNA metabolism, reinforced the involvement of SEPs with DNA metabolism seen in Figure 6B. For KEGG pathway, SEP number surpassed canonical proteins in Prokaryotic defense system (BR:ko02048) [42]. Most of these SEPs (~99.4%) possessed the reverse transcriptase domain and might located in retrotransposons, hinting the historical events on integration of virus genes into the human genome [43]. The remaining 58 SEPs involved in this pathway were similar with the single-copy and essential canonical proteins, including methyltransferase DNMT1 and transfer RNA synthetase EARS2.

## CONCLUSION

In this work, we developed a framework to predict the coding potential of smORF named sOCP. The framework starts with constructing a model through data collection, model selection and feature selection to optimize its performance and is applied in predicting novel smORFs from the human genome. Various complicated features have been used in sequence-based prediction. However, our research implies that a simplified model based on TIS and in-frame features for a limited dataset size is efficient enough to judge if the smORF candidate will be translated. The evaluation of the heterogenous OpenProt Altprots with experimental evidence further proved the value of our model. The main limitation is that the prediction depends on known coding and non-coding sequence data. Small coding ORFs demand evidence from translatomic and proteomic studies. Thus, a species with little data might be unable to afford a training dataset large enough to construct a credible model. As more and more smORFs among various species are experimentally validated, the method integrating intrinsic feature-based classifiers and alignment-based analysis is expected to have a significant advantage on smORF prediction.

The sOCP model could be applied to screen novel smORFs from the human genome, with high coding potential predicted using our model. The novel human smORF database includes ~400k smORFs started with ATG and 700k smORFs with non-ATG start codons and will help further translatomic and proteomic studies for database search. Annotation of the novel SEPs presumed that they bear functions seldomly seen in canonical proteins and re-emphasized the noteworthy ‘dark proteome’ poorly understood currently.

**Key Points**

- Developed the sOCP pipeline applied in smORF prediction from genomes.
- Indicated the importance of TIS and in-frame features for prediction of smORF coding potential.
- Generated a database providing novel smORFs and microproteins in human.

## Data Availability

The source code of sOCP is available at https://github.com/bite123/sOCP. The human smORF database generated in this study are available in FigShare with the DOI 10.6084/m9.figshare.24707838.

## Author Biographies

**Zhao Peng** is a post-doctor in School of Life Sciences, Central China Normal University. His research interests include evolutionary genomics and peptidomics.

**Jiaqiang Li** is a master student in School of Computer Science, Central China Normal University. His research interests include bioinformatics and machine learning.

**Xingpeng Jiang** is a professor in School of Computer Science, Central China Normal University. His research interests include artificial intelligence, large data mining and high-dimensional data analysis.

**Cuihong Wan** is a professor in School of Life Sciences, Central China Normal University. Her research interests include mass spectrometry analysis, peptidomics and protein complex evolution.
